# Seven New Complete Plastome Sequences Reveal Rampant Independent Loss of the *ndh* Gene Family across Orchids and Associated Instability of the Inverted Repeat/Small Single-Copy Region Boundaries

**DOI:** 10.1371/journal.pone.0142215

**Published:** 2015-11-11

**Authors:** Hyoung Tae Kim, Jung Sung Kim, Michael J. Moore, Kurt M. Neubig, Norris H. Williams, W. Mark Whitten, Joo-Hwan Kim

**Affiliations:** 1 Department of Life Science, Gachon University, Seongnam, Gyeonggi-do, Korea; 2 Department of Biology, Oberlin College, Oberlin, Ohio, United States of America; 3 Florida Museum of Natural History, University of Florida, Gainesville, Florida, United States of America; University of Naples Federico II, ITALY

## Abstract

Earlier research has revealed that the *ndh* loci have been pseudogenized, truncated, or deleted from most orchid plastomes sequenced to date, including in all available plastomes of the two most species-rich subfamilies, Orchidoideae and Epidendroideae. This study sought to resolve deeper-level phylogenetic relationships among major orchid groups and to refine the history of gene loss in the *ndh* loci across orchids. The complete plastomes of seven orchids, *Oncidium sphacelatum* (Epidendroideae), *Masdevallia coccinea* (Epidendroideae), *Sobralia callosa* (Epidendroideae), *Sobralia* aff. *bouchei* (Epidendroideae), *Elleanthus sodiroi* (Epidendroideae), *Paphiopedilum armeniacum* (Cypripedioideae), and *Phragmipedium longifolium* (Cypripedioideae) were sequenced and analyzed in conjunction with all other available orchid and monocot plastomes. Most *ndh* loci were found to be pseudogenized or lost in *Oncidium*, *Paphiopedilum* and *Phragmipedium*, but surprisingly, all *ndh* loci were found to retain full, intact reading frames in *Sobralia*, *Elleanthus* and *Masdevallia*. Character mapping suggests that the *ndh* genes were present in the common ancestor of orchids but have experienced independent, significant losses at least eight times across four subfamilies. In addition, *ndhF* gene loss was correlated with shifts in the position of the junction of the inverted repeat (IR) and small single-copy (SSC) regions. The Orchidaceae have unprecedented levels of homoplasy in *ndh* gene presence/absence, which may be correlated in part with the unusual life history of orchids. These results also suggest that *ndhF* plays a role in IR/SSC junction stability.

## Introduction

With over 25,000 species, Orchidaceae are one of the two largest families of angiosperms, exceeded only perhaps by Asteraceae [1]. Although molecular phylogenetic analyses over the past two decades have brought much of the deeper-level relationships of Orchidaceae into focus [2–6], these studies have relied primarily on ITS and a handful of plastid loci such as *trnL-F* and *ycf1* ([7] and references therein), and many deep-level relationships (e.g., among tribes of Epidendroideae) remain unclear. The potential of whole plastome sequences for resolving relationships of orchids is as yet only poorly explored [8].

Five monophyletic subfamilies are now recognized in Orchidaceae, with the following relationships: (Apostasioideae, (Vanilloideae, (Cypripedioideae, (Orchidoideae, Epidendroideae)))) [7]. Apostasioideae (*Apostasia* Blume and *Neuwiedia* Blume) are sister to all other orchids and are divergent in molecular data and flower structure [9]. Vanilloideae consist of 15 genera and about 180 species, belonging to the tribes Pogonieae and Vanilleae. Cypripedioideae show a number of synapomorphies such as a deeply saccate labellum, two fertile stamens, a shield-like staminode, and a synsepal composed of the fused lateral sepals. The subfamily is composed of five genera including 176 species that are found in a variety of habitats [10, 11]. The remaining two subfamilies, Orchidoideae and Epidendroideae, comprise the bulk of species richness in the family. Orchidoideae are comprised of 208 genera and four tribes—Codonorchideae, Cranichideae, Diurideae, and Orchideae [7]. Epidendroideae is the largest subfamily in Orchidaceae, with approximately 659 genera, many of which have greatly changed in generic circumscription over recent years. Sixteen tribes are recognized within Epidendroideae in the most recent revision of Orchidaceae classification [7].

The plastome of land plants generally contains 30–50 different RNA genes and about 100 protein-coding genes [12]. It is highly conserved in gene order and content with a typical circular form [13]. Data from complete plastomes are now widely utilized in phylogenetic studies of plants, where they have helped resolve deep-level relationships among major lineages and have revealed patterns of plastome structural evolution, including rare but extensive rearrangements and gene loss, as for example the loss of the entire *ndh* gene family [8, 14–24].

Early attempts at sequencing *ndhF* in orchids revealed that the gene has been pseudogenized or lost in numerous orchid taxa [6, 25], causing orchid systematists to abandon *ndhF* as a phylogenetic marker in favor of other plastid genes such as *rbcL* and *matK*. Subsequent complete plastome sequences of various photosynthetic and non-photosynthetic orchids revealed that all 11 *ndh* loci were lost or pseudogenized in nearly all of the plastomes [8, 14, 26–36], suggesting that the loss of *ndh* loci occurred early in the evolutionary history of the family. However, recent work has documented the presence of full-length plastid copies of all *ndh* loci in several orchid lineages, including *Masdevallia picturata* Rchb.f. [8], *Calanthe triplicata* (Willemet) Ames [37] (Epidendroideae), *Goodyera fumata* Thwaites [8], *Habenaria pantlingiana* Kraenzl. [8] (Orchidoideae), *Cypripedium formosanum* Hayata [8], and *Cypripedium japonicum* Thunb. [23] (Cypripedioideae), and *Apostasia wallichii* R. Br. (Apostasioideae) [19]. These results strongly imply that the *ndh* genes were present in the common ancestor of Orchidaceae but have been lost independently in multiple lineages of orchids [8].

The *ndh* genes encode subunits of the NADH dehydrogenase-like complex, which mediates cyclic electron flow around Photosystem I and facilitates chlororespiration [8, 38], but they have been found to be dispensable for plant growth under optimal growth conditions [39]. Using complete plastome sequencing, the *ndh* loci have also been found to be absent from several other lineages of land plants, particularly from groups with unusual trophic status. For example, the *ndh* loci are absent from parasitic plants such as Orobanchaceae and *Cuscuta* (Convolvulaceae) [17, 40, 41], from mycoheterotrophs such as *Petrosavia* (Petrosaviaceae) [42], and from plastomes that have undergone extensive rearrangement such as *Erodium* (Geraniaceae) [43]. The *ndh* loci have also been lost from other photosynthetic seed plants as well, such as Gnetales, conifers, and *Najas* (Hydrocharitaceae) [44–47].

To date, 32 complete orchid plastomes have been reported for Orchidaceae [8, 14, 26–37]. All but eight of these plastomes [the exceptions being one Vanilloideae, four Cypripedioideae and three Orchidoideae plastomes] are from Epidendroideae, one of the most species-rich and diverse subfamilies that includes many epiphytic and some terrestrial and mycoheterotrophic orchids. Additional plastome data from a broader phylogenetic sampling of subtribes is essential to understand the evolutionary history of orchids and the orchid plastome. Here we describe and analyze the complete plastomes of seven orchids, with a focus on characterizing patterns of *ndh* gene family retention and loss across Orchidaceae. We also document a correlation between the loss of *ndh* loci (particularly *ndhF*) and the positional instability of the inverted repeat (IR)/small single-copy (SSC) region boundary. To contextualize patterns of *ndh* gene loss and to refine estimates of phylogenetic relationships among orchids, we extracted all 79 protein-coding genes from these seven plastomes and all other currently sequenced orchid plastomes, and included them in a phylogenetic analysis of 117 angiosperm taxa, including all available monocot plastomes.

## Materials and Methods

### Taxon sampling, DNA extraction and sequencing

We sequenced the complete plastomes of the following seven orchid species: *Oncidium sphacelatum* Lindl. (*W*. *M*. *Whitten 3467*, Epidendroideae: Cymbidieae), *Masdevallia coccinea* Linden ex Lindl. (*W*. *M*. *Whitten 3569*, Epidendroideae: Epidendreae), *Sobralia callosa* L.O.Williams (*W*. *M*. *Whitten 3275*, Epidendroideae: Sobralieae), *Sobralia* aff. *bouchei* (*K*. *Neubig 208*, Epidendroideae: Sobralieae), *Elleanthus sodiroi* Schltr. (*K*. *Neubig 246*, Epidendroideae: Sobralieae), *Paphiopedilum armeniacum* S.C. Chen & F.Y.Liu (*W*. *M*. *Whitten 3315*, Cypripedioideae) and *Phragmipedium longifolium* (Warsz. & Rchb.f.) Rolfe (*W*. *M*. *Whitten 2804*, Cypripedioideae). All species were sampled from greenhouse material at the University of Florida, and were originally acquired from botanical gardens under CITES permits except for *P*. *armeniacum*, which was sampled under USDA/APHIS authorization from material rescued from illegal importation. Voucher specimens were deposited in the herbarium of the Florida Museum of Natural History (FLAS). A total of 116 plastome sequences, including six basal angiosperm outgroups, as well as the plastid coding regions of *Apostasia wallichii* (74 genes, HQ180402–HQ183419) were downloaded from GenBank for inclusion in phylogenetic analyses (S1 Table), resulting in a final matrix of 124 taxa.

The plastomes of *Elleanthus sodiroi* and *Paphiopedilum armeniacum* were sequenced using a genome skimming approach [48] on an Illumina MiSeq, yielding 28,226,982 and 24,626,728 reads, respectively. Assemblies of both plastomes were performed in Geneious 7.1.8 (Biomatters Ltd., Auckland, New Zealand). Read ends were trimmed with an error probability limit of 0.01 (i.e. end regions with more than 1% chance of an error per base were trimmed). For each library, reads were aligned to previously sequenced orchid plastomes using the Geneious assembler with medium-low sensitivity. Assembled reads were then *de novo* assembled using the Geneious assembler, with zero mismatches and gaps allowed among reads. The reads were then re-aligned to the resulting *de novo* contigs with zero mismatches and gaps, with 100 iterations. The resulting contigs were then *de novo* assembled, using the circularize contigs and matching ends options. The remaining five plastomes were sequenced from chloroplast isolations on a 454 GS FLX at the University of Florida Interdisciplinary Center for Biotechnology Research. Chloroplast isolation, sequencing, assembly, and Sanger-based gap closure for these five plastomes followed the protocols outlined in [49, 50].

Plastomes were annotated using DOGMA [51], Geneious 7.1.8 [52], and tRNAscan-SE [53], with comparisons to all published orchids and other monocot plastid genomes. The exact positions of genes were further confirmed using local BLAST searches against representative monocot plastomes (including orchids) deposited in the NCBI Genome database. Summary statistics for sequencing and plastome characteristics for the seven newly reported plastomes are provided in Table 1.

**Table 1:** Summary statistics for the seven newly generated orchid plastomes.

	*Elleanthus sodiroi*	*Masdevallia coccinea*	*Oncidium sphacelatum*	*Paphiopedilum armeniacum*	*Phragmipedium longifolium*	*Sobralia* aff. *bouchei*	*Sobralia callosa*
**Sequencing platform**	MiSeq	454 GS FLX	454 GS FLX	MiSeq	454 GS FLX	454 GS FLX Titanium	454 GS FLX Titanium
**Number of plastid reads**	237,687	22,498	7,689	383,904	22,018	44,795	97,492
**Average coverage**	296.7x	32.9×	12.0×	308.1x	33.5×	161×	211×
**Total length (bp)**	161,511	157,423	147,761	162,682	151,157	161,543	161,430
**LSC (bp)**	88,425	84,957	83,575	91,942	88,367	88,684	88,666
**IR (bp)**	27,103	27,009	25,758	33,536	24,862	27,012	26,985
**SSC (bp)**	18,880	18,448	12,670	3,668	13,066	18,835	18,794
**GC %**	37.1	36.8	37.1	35.4	36.1	37.1	37.1
**Coding region %**	56.5	57.9	53.3	56.1	52.4	56.5	56.5
**Intron region % (including *rps12* intron 2)**	12.8	12.8	11.6	11.2	12.3	12.9	12.9
**Intergenic spacer %**	30.7	29.4	35.1	32.7	35.3	30.6	30.7

LSC, large single-copy region; IR, inverted repeat; SSC, small single-copy region

### Phylogenetic analyses

The sequences of all 79 plastid protein-coding genes (S2 Table) were extracted from all 124 plastomes and were translated and aligned with MAFFT [54] using the following parameters: genetic code = standard, translation frame = 1, scoring matrix = BLOSUM62, and gap open penalty = 1.53.

The GTR + Γ and GTR + I + Γ models were compared using PartitionFinder v1.1.1 [55, 56] for the total data set partitioned by gene. Maximum likelihood (ML) analyses were performed on the unpartitioned data set as well as the partitioned data using RAxML Black Box [57, 58] as implemented in the CIPRES SCIENCE Gateway [59]. For both ML analyses, 100 bootstrap replicates were completed using RAxML. Bayesian analysis was performed using MrBayes [60] under the GTR + I + Γ model (ngen = 1,000,000, samplefreq = 200, burninfrac = 0.25) based on the unpartitioned data. The average standard deviation of split frequencies was used to determine whether stationarity was reached among replicates.

### Character reconstructions of *ndh* gene status in Orchidaceae

For all 42 Asparagales plastomes in our data set, all *ndh* genes were scored as belonging to one of the following four classes: 1) present (i.e., full-length and in frame), 2) pseudogenized (i.e., stop codons induced by small frame shifts), 3) truncated (> 10% of the typical gene length deleted) and 4) completely deleted. To explore the effects of different patterns of gene loss across the *ndh* loci, we created two different character step matrices for parsimony optimization. In the first step matrix, the four character states were treated as a directional series from 1 → 4, such that any character state change in the “forward” direction (e.g., from 1 → 2, or from 3 → 4, or from 1 → 4) required only 1 step, whereas the second step matrix incorporated a progressive series from 1 → 2 → 3 → 4; i.e., complete gene loss required first progressing through pseudogenization and truncation. Because reversals from a non-functional or deleted *ndh* gene (states 2–4) to a functional, in-frame gene (state 1) are unlikely due to the fact that horizontal gene transfer is essentially unknown in angiosperm plastomes [14], any change in the “reverse” direction required 5 steps in either step matrix. In this manner, reversals were heavily penalized but not impossible. All character state reconstructions were performed in Mesquite v. 3.02 (build 681) [61] using the best ML tree topology.

## Results

### Phylogenetic relationships

**Fig 1:**
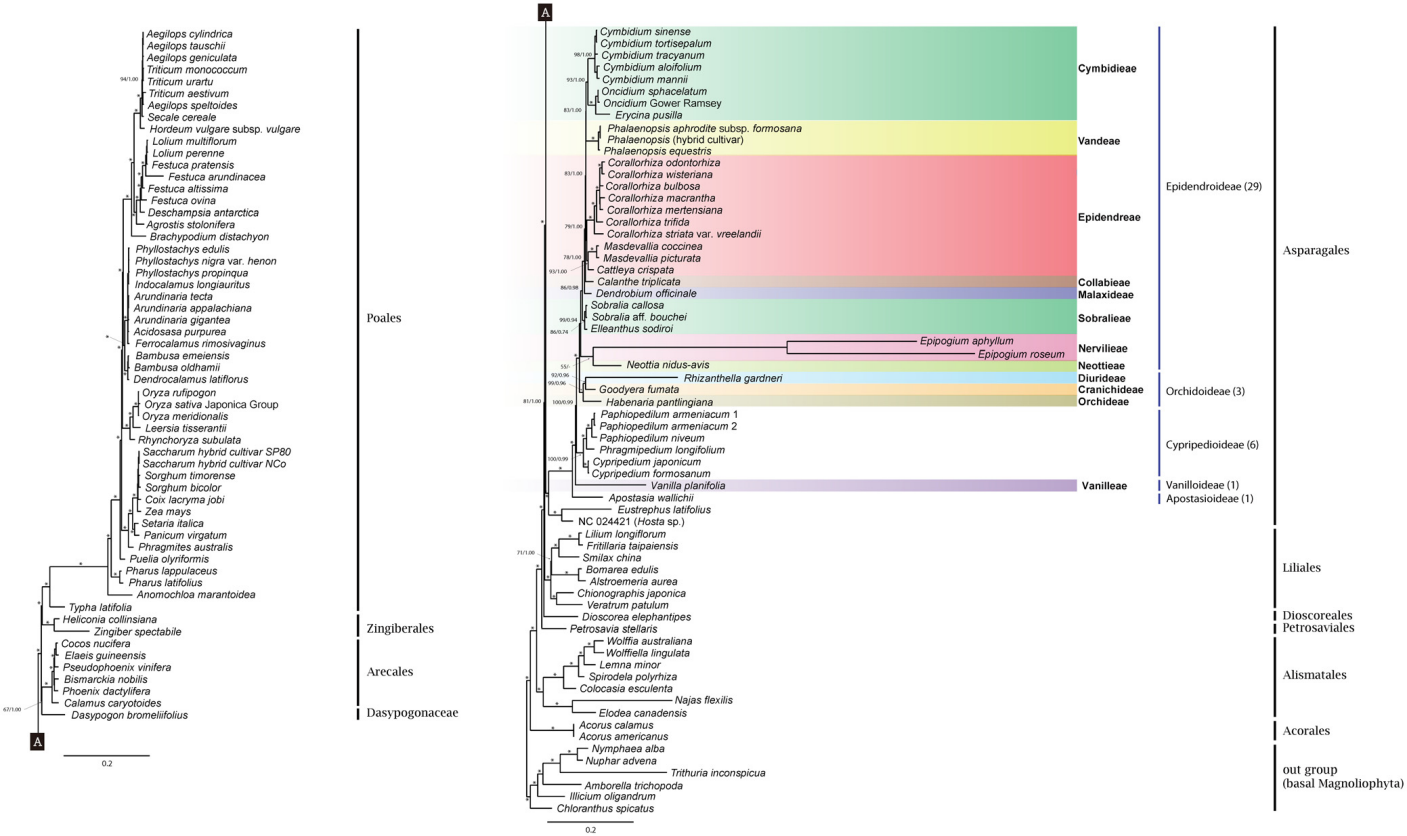
Maximum likelihood tree of monocots generated from 79 plastid coding gene sequences (based on both partitioned and unpartitioned data), with support values indicated. An asterisk on a branch means that it was supported by 100% bootstrap value (BP) in the RAxML trees and a Bayesian posterior probability of 1.00.

The final data set consisted of 84,414 characters, of which 43,746 characters were constant and parsimony-uninformative, 12,162 characters were variable and parsimony-uninformative, and 28,506 characters were parsimony-informative. ML (including partitioned and unpartitioned analyses) and Bayesian analyses recovered the same tree topology (Fig 1). The monophyly of all orders in the monocots was strongly supported (Fig 1). Asparagales were sister to the clade of Poales + Zingiberales + Arecales + Dasypogonaceae. Within the Asparagales, the Orchidaceae were strongly supported as monophyletic and were sister to a strongly supported clade of *Eustrephus latifolius* R.Br. (Asparagaceae) and the anomalously placed *Cypripedium macranthos* Sw. (Orchidaceae; RefSeq accession number NC_024421). In response to the anomalous placement of *C*. *macranthos*, we conducted BLAST searches of *matK*, *ndhF*, and *rbcL* from NC_024421, which revealed a high sequence similarity (> 99.7% identity) to sequences from species of *Hosta* Tratt. (Asparagaceae). These data suggest that the NCBI complete plastome for *C*. *macranthos* is likely incorrectly labeled taxonomically. Consequently, “*C*. *macranthos*” was treated as a member of Asparagaceae in downstream analyses and was relabeled with the RefSeq accession number for the purposes of the present study.

Relationships within Orchidaceae were also generally well-supported (Fig 1). The monophyly of all subfamilies and tribes was well supported in the ML and Bayesian trees, with only two branches in the family receiving < 100% bootstrap and < 1.0 Bayesian posterior probability (Fig 1): the branch uniting Epidendroideae (15% / 0.89), and the branch uniting Vandeae and Cymbideae (88% / 1.0).

### Plastome structure and *ndh* loss

**Fig 2:**
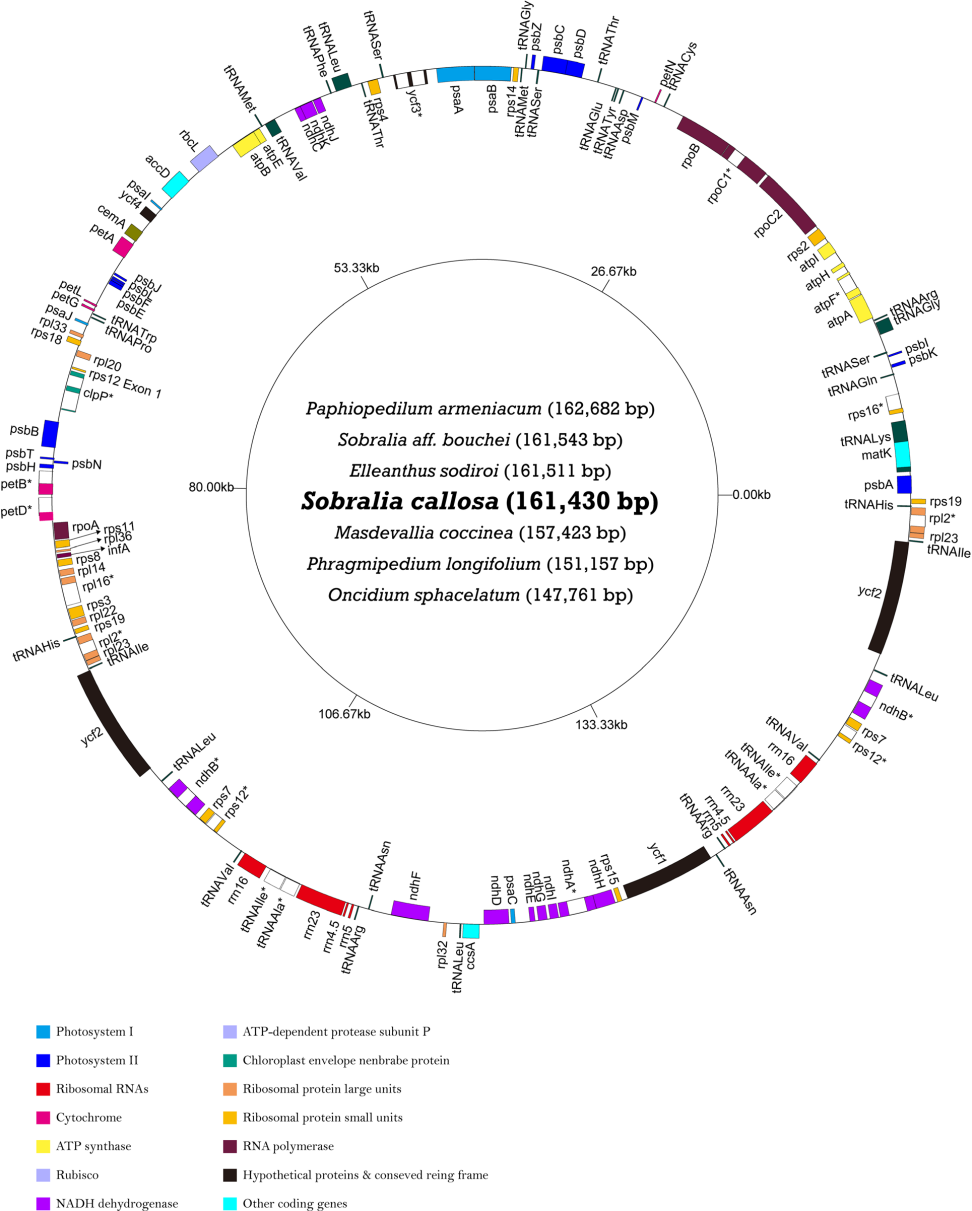
Map of the seven newly sequenced orchid plastomes. Genes are distinguished by colors based on their function. An asterisk indicates a gene with an intron. The direction of transcription is clockwise for genes on the inside of the circle, and counterclockwise for genes on the outside.

**Fig 3:**
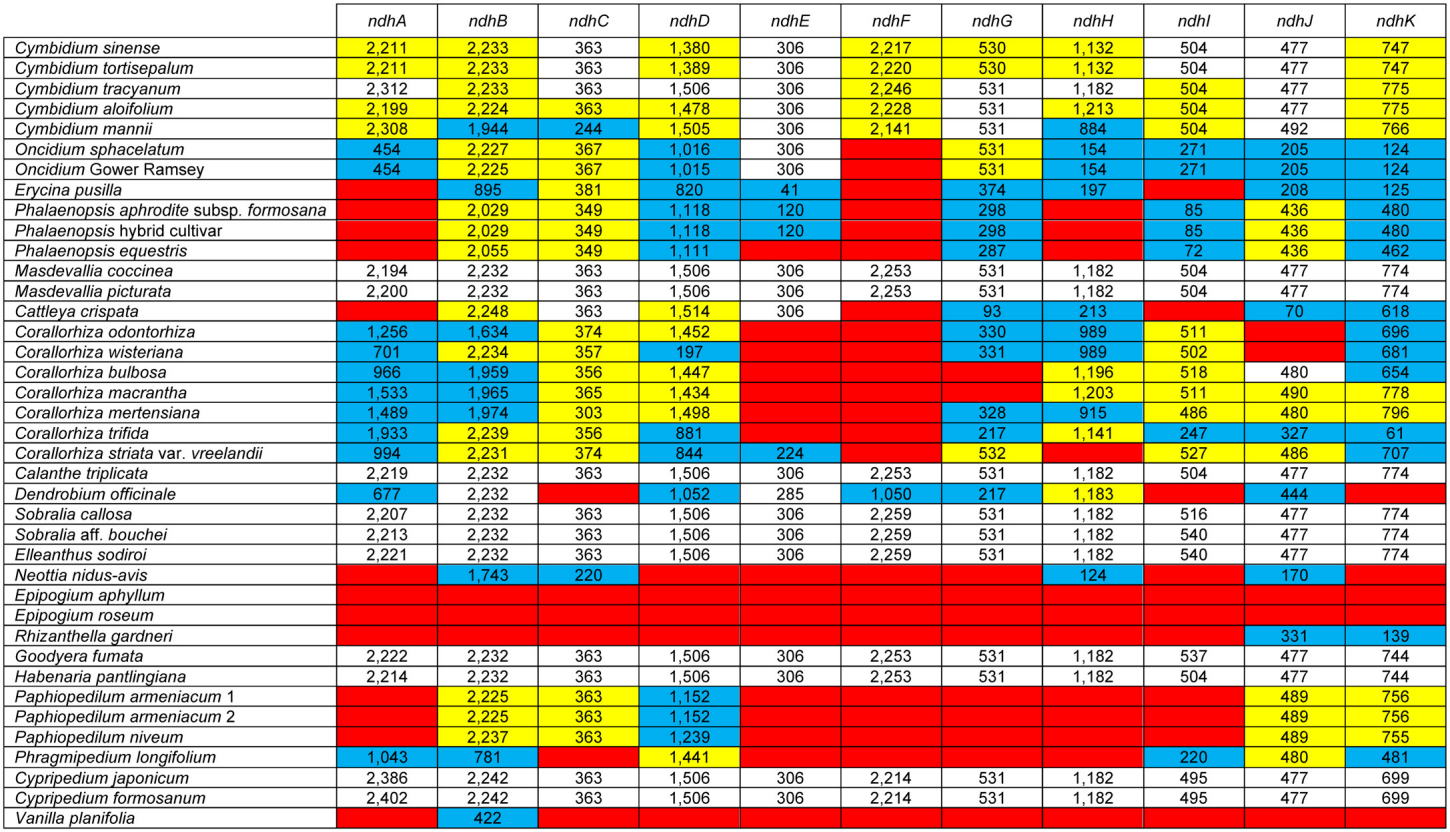
*ndh* gene length variation among orchid plastomes. Numbers within cells refer to gene or pseudogene lengths. Key to colored cells: white—full-length, in frame; yellow—pseudogenized (very short, frame-shift inducing indels); blue—truncated (significant indels); red—completely deleted. The lengths of *ndhA* and *ndhB* include their introns. Taxa are presented in the same order as in Fig 4. Gene lengths are not provided for *Apostasia wallichii* because in some cases, small portions of the 5′ or 3′ ends of the genes were not sequenced and were hence unavailable in GenBank; likewise, only coding sequences were available (no introns were included) for the two intron-containing *ndh* loci [19].

All seven newly sequenced orchid plastomes were found to possess the typical quadripartite angiosperm plastome organization and gene arrangement (Fig 2). Summary statistics for each plastome are provided in Table 1. The total length of the seven plastomes varied from 147.7 kb to 162.7 kb, with differences in total length largely due to differences in IR length and the presence or absence of *ndh* loci (Table 1, Fig 3). The effect of *ndh* gene loss on overall length was most pronounced in the small single-copy (SSC) regions, where 7 of 11 *ndh* genes are located. The GC content of these plastomes varied from 35.4 to 37.1% (Table 1). Gene content among the genomes was the same except with respect to the *ndh* gene family. All *ndh* genes possessed full reading frames in *Sobralia callosa*, *Sobralia* aff. *bouchei*, *Elleanthus sodiroi* and *Masdevallia coccinea*. In contrast, the *ndh* genes exhibited varying degrees of loss in *Oncidium sphacelatum*, *Paphiopedilum armeniacum* and *Phragmipedium longifolium*, with > 50% of the total combined length of all 11 *ndh* loci lost in each of these three taxa (Fig 3). In the *O*. *sphacelatum* plastome, only *ndhE* possessed a full reading frame; the *ndhF* locus was deleted and all other *ndh* genes were either pseudogenized and/or possessed significant indels, suggestive of functional gene loss. Five and six of the *ndh* genes were found to be deleted in the *P*. *armeniacum* and *P*. *longifolium* plastomes, respectively. All other *ndh* loci in these two plastomes were pseudogenized or truncated by significant indels (Fig 3).

**Fig 4:**
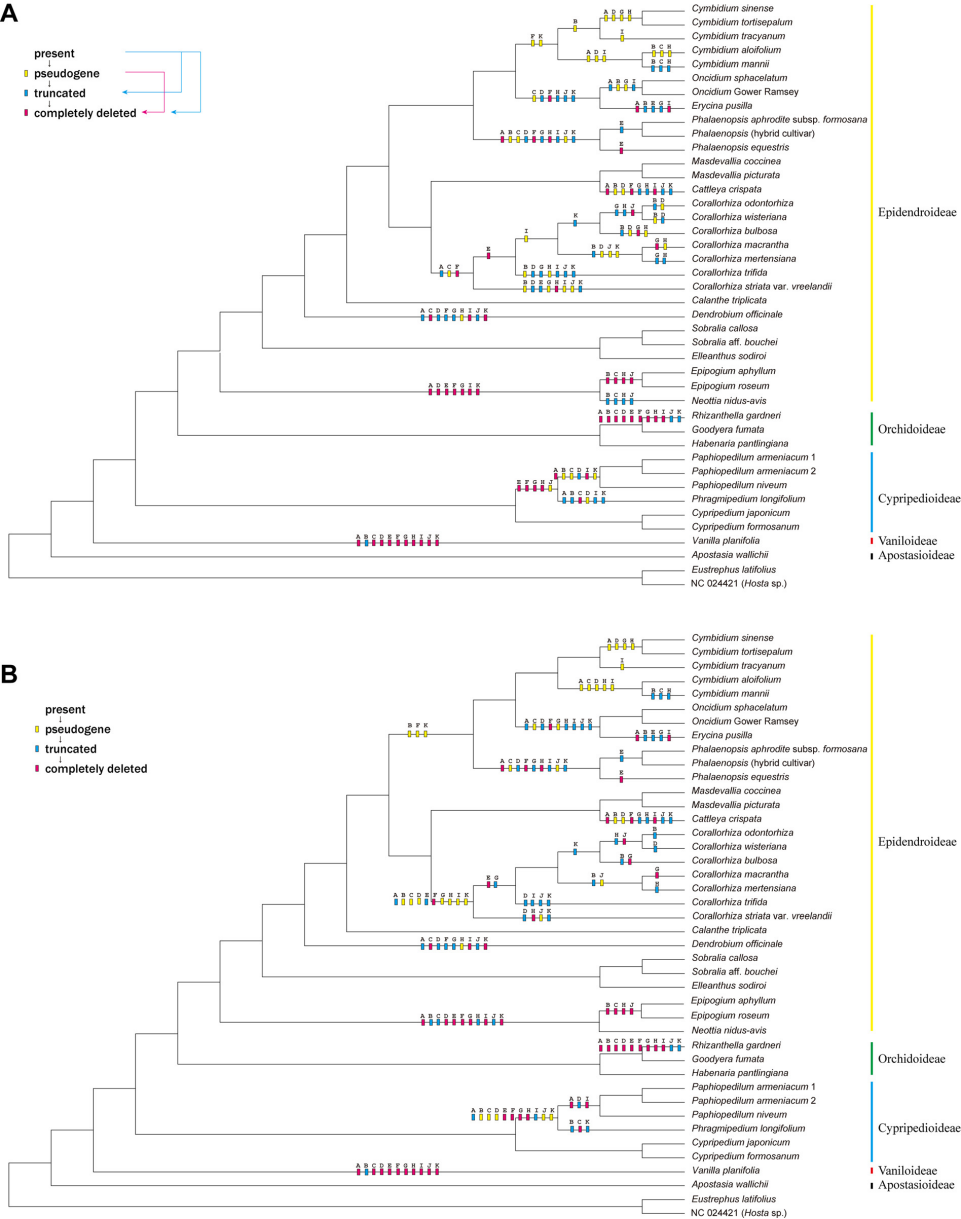
Character reconstructions of *ndh* gene loss and pseudogenization events, mapped onto the Orchidaceae portion of the ML tree from Fig 1. The insets show graphical representations of the step matrices used in the character reconstructions; see text for details. Taxa are presented in the same order as in Fig 3.

Character state reconstructions of *ndh* gene status across orchids revealed a complex pattern of independent gene loss and pseudogenization (Fig 4). The two alternative reconstructions of gene loss did not differ significantly in their overall patterns, although minor differences in the positions of some events were inferred in the clade of *Cymbidium*, *Oncidium*, *Erycina*, and *Phalaenopsis*. The total number of required changes was substantially lower (steps across all 11 *ndh* loci) under the first step matrix (156 steps, Fig 4A) compared to the second step matrix (261 steps; Fig 4B).

No evidence of loss or pseudogenization at any *ndh* locus was detected in 10 of the 39 orchid plastomes in our data set, including all taxa of *Cypripedium*, *Calanthe*, *Habenaria*, *Goodyera*, *Elleanthus*, *Sobralia*, and *Masdevallia*. In addition, no loss or pseudogenization was evident in the 11 individual *ndh* gene sequences for *Apostasia walllichii*; however, it is important to note that only coding sequences have been submitted to GenBank for this taxon, and in some cases small portions of the 5′ or 3′ ends of the *ndh* genes are missing because they were not sequenced and were hence unavailable for our analyses. Phylogenetically, these 11 taxa that displayed no evidence of *ndh* loss or pseudogenization were scattered throughout Orchidaceae and included representatives of four of the five subfamilies of orchids (Fig 4).

In contrast, all or nearly all *ndh* loci were found to have been deleted, truncated, and/or pseudogenized in all other orchid plastomes in our data set. Character state optimization revealed that the *ndh* gene family was present in the ancestor of Orchidaceae but experienced independent, significant losses (where loss is defined as possessing a nucleotide sequence indicative of producing a non-functional protein, or no protein) a minimum of eight times in Orchidaceae, and perhaps as many as ten times: (1) in the ancestor of *Cymbidium*, *Oncidium*, *Erycina*, and *Phalaenopsis* [the reconstructions under step matrix 1 (Fig 4A) suggest the possibility of three independent losses of the *ndh* loci in this larger clade, specifically in the ancestor of *Cymbidium*, the ancestor of *Erycina* and *Oncidium*, and the ancestor of *Phalaenopsis*]; (2) in *Cattleya*; (3) in the ancestor of *Corallorhiza*; (4) in *Dendrobium*; (5) in the ancestor of *Epipogium* and *Neottia*; (6) in *Rhizanthella*; (7) in the ancestor of *Paphiopedilum* and *Phragmipedium*; and (8) in *Vanilla*. In all eight of these instances of *ndh* loss, four or more of the *ndh* genes have experienced significant deletions or total loss (Fig 4). The mycoheterotrophic orchids *Epipogium*, *Neottia* and *Rhizanthella* experienced the most extreme *ndh* gene losses, with all *ndh* genes lost in *Epipogium* and only two and four partially remaining *ndh* loci in *Rhizanthella* and *Neottia*, respectively. In contrast, the least extreme *ndh* losses were evident in *Cymbidium*, in which all or nearly all genes were pseudogenized; i.e. no or only a handful of significant deletions were detected (Fig 4).

**Fig 5:**
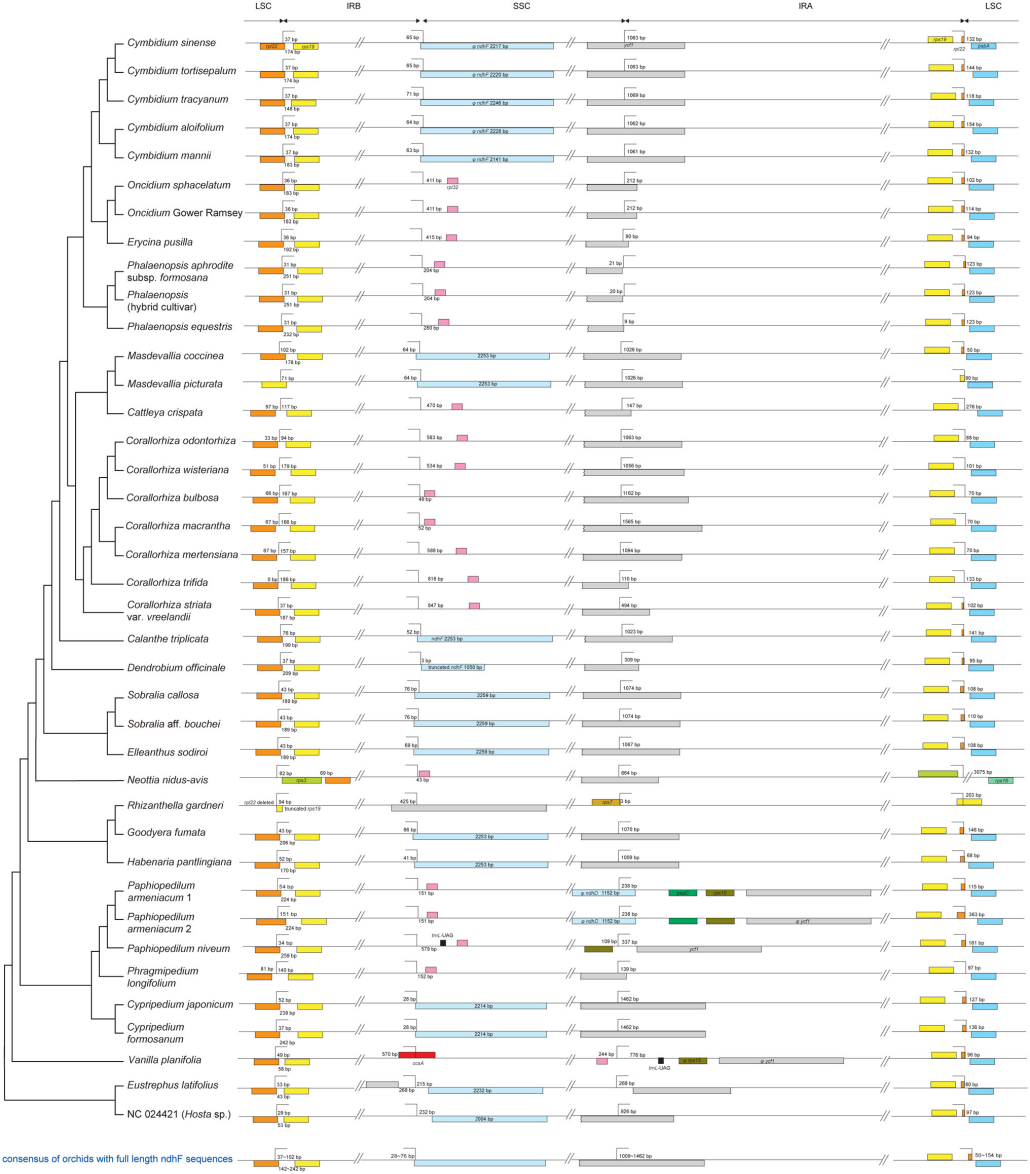
Comparison of IR boundaries among 37 orchid plastomes and Asparagaceae outgroups. The complete *Apostasia* plastome is unpublished and hence unavailable for comparison, and the IRs in the two complete *Epipogium* plastomes are highly truncated along with the rest of the plastome (total plastome length of 30.7 kb in *E*. *aphyllum* and 19.0 kb in *E*. *roseum*), and thus are not shown here.

We compared the locations of the IR/single-copy region junctions among 37 orchid plastomes and those of the two most closely related taxa of Asparagales (Fig 5). The complete *Apostasia wallichii* plastome is unpublished and hence was unavailable for comparison, and the IRs in the two complete *Epipogium* plastomes are highly truncated along with the rest of the plastome (total plastome length of 30.7 kb in *E*. *aphyllum* and 19.0 kb in *E*. *roseum*) [62], and thus were not included in comparisons. Within Orchidaceae, all plastomes with full, in-frame copies of *ndh* loci as well as those with the least amount of *ndh* loss (i.e., *Cymbidium*) possessed the typical approximate IR boundaries observed in angiosperms; for example, in all cases, the SSC/IR_B_ junction was located at the extreme 3′ end of *ndhF*, whereas the SSC/IR_A_ junction was located approximately 1 kb downstream of the 5′ end of *ycf1*. In contrast, substantial variation in the positions of the SSC/IR junctions was observed in taxa with more advanced degrees of *ndh* loss (Fig 5). In nearly all cases of independent loss of *ndhF*, the SSC/IR_B_ junction was located in the spacer region adjacent to *rpl32*, ranging from 43 to 847 bp away from the start of *rpl32*. In almost all cases of severe truncation or loss of *ndhF*, the SSC/IR_A_ junction experienced a shift toward the 5′ end of *ycf1* or to a highly anomalous position entirely outside of *ycf1* (the latter position was observed in *Vanilla*, *Rhizanthella*, and *Paphiopedilum*; Fig 5). In most instances, this shift was substantial: movement of the SSC/IR_A_ junction to a position within 300 bp of the start of *ycf1* was common, and in *Phalaenopsis* the junction shifted to a position just upstream of *ycf1* (Fig 5). Only in some species of *Corallorhiza* was a shift in the SSC/IR_B_ toward the start of *ycf1* detected in association with the loss of *ndhF*, and even in these instances the position of the SSC/IR_B_ junction varied by 400 bp among species. The shift of the SSC/IR_A_ junction was most dramatic in *Paphiopedilum*. Here the IR expanded to include all of φ*ycf1*, *rps15*, *psaC*, and φ*ndhD*, resulting in an anomalously large 33.5 kb IR. A similar set of unusual IR boundary shifts occurred in *Vanilla*, leading to all of φ*ycf1*, φ*rps15*, *trnL-UAG*, and a portion of *ccsA* being included in the IR (Fig 5).

In contrast to the instability of the IR/SSC boundaries in *ndh*-lacking taxa, the IR/LSC boundaries were found to be relatively stable (Fig 5). In almost all orchids, the LSC/IR_B_ junction was found to be near the start of *rpl22*, the only exceptions being the highly reduced plastomes of *Rhizanthella* (in which *rpl22* is deleted) and *Neottia*, and in *Masdevallia picturata*, in which the junction has shifted to near the start of *rps19* (Fig 5). The LSC/IR_A_ junction also occupied its typical monocot position near the 3′ end of *psbA* in all orchids except the mycoheterotrophic *Neottia* and *Rhizanthella* (Fig 5).

## Discussion

### Phylogenetic relationships within Orchidaceae

Our phylogenetic analyses reveal strong support for nearly all branches of Orchidaceae, most notably along the backbone of Epidendroideae, which has not been strongly supported in previous analyses based on fewer genes (e.g., [63]). Only the placement of *Phalaenopsis* as sister to Cymbideae has less than full bootstrap and Bayesian support (Fig 1). The relationships among all of the subfamilies and many of the tribes of Orchidaceae included in our analyses are largely congruent with recent studies [7], although a handful of differences are evident in tribal relationships. Neottieae and Sobralieae are successively sister to remaining Epidendroideae, in agreement with previous studies (e.g., [2, 9, 63]). However, the position of Epidendreae as sister to Vandeae + Cymbidieae in the plastome phylogeny (Fig 1) differs from its position in previous phylogenies. For example, in the 7-locus phylogeny of Freudenstein and Chase [63], Epidendreae was sister to Cymbidieae but with only weak support, whereas in Chase *et al*. [7] it is placed as sister to Vandeae. The differences observed between previous studies and our own are likely due to the great disparities in number of characters and taxa included [64]. Even though our matrix is character-rich and hence may be less prone to error induced by individual genes [65], we must emphasize that our taxon sampling is highly incomplete, and it is possible that additional plastome sequences from poorly sampled and/or unsampled tribes and subtribes may result in topological changes. Moreover, the pseudogenization and/or absence of numerous loci from the mycoheterotrophic orchids *Corallorhiza*, *Epipogium*, *Neottia*, and *Rhizanthella* [28–30] result in relatively large amounts of missing data for these taxa. The addition of more closely related photosynthetic relatives of these lineages may also influence the positions of major orchid groups, although we emphasize that their positions in the current tree are congruent with their positions in previous analyses.

### *ndh* gene deletions and IR boundaries in Orchidaceae

Among 84 currently available non-Orchidaceae monocot plastomes, only two (*Najas flexilis* and *Petrosavia stellaris*) have lost all *ndh* genes. These losses in the plastome of *Najas flexilis* have been suggested to result from adaptation to submerged environments [47] and those of *Petrosavia stellaris* [42] are attributable to its nonphotosynthetic lifestyle. In contrast to the rest of monocots, *ndh* gene losses have been frequent throughout Orchidaceae, and have occurred in both nonphotosynthetic and photosynthetic lineages.

Our results strongly imply that the ancestral orchid plastome possessed full ORFs for all *ndh* genes and that, surprisingly, the *ndh* gene family has experienced at least eight independent, significant losses throughout the Orchidaceae, occurring across at least four of the five subfamilies (Vanilloideae, Cypripedioideae, Orchidoideae and Epidendroideae). The seemingly chaotic pattern of pseudogenization, deletion, and truncation of the *ndh* loci among orchids (Fig 5) further supports our interpretation of homoplastic loss of this gene family at differing times in different lineages. For example, among the achlorophyllous, mycoheterotrophic orchids, the deletion of *ndh* loci is more advanced in *Epipogium*, *Neottia* and *Rhizanthella* compared to *Corallorhiza*, suggesting that perhaps the loss of these loci occurred earlier in the ancestor of these genera than it did in the ancestor of *Corallorhiza* [30]. In addition, the gene losses of *Corallorhiza* with non-visible green tissue (*C*. *macrantha*, *C*. *mertensiana* and *C*. *striata* var. *vreelandii*) were not significantly higher than those of *Corallorhiza* with at least some visible green tissue (*C*. *odontorhiza*, *C*. *wisteriana*, *C*. *bulbosa* and *C*. *trifida*), suggesting that gene loss may not be entirely correlated with loss of photosynthesis in *Corallorhiza*.

Among photosynthetic orchids, patterns of gene loss are also inconsistent, both across the Orchidaceae and at lower taxonomic levels. For example, among lineages that have lost all or most of the *ndh* loci, *ndhE* has been truncated or deleted in *Erycina*, *Phalaenopsis*, *Paphiopedilum*, *Phragmipedium* and *Vanilla* but is full length in *Oncidium*, *Cymbidium* and *Cattleya*, and is also in frame (although 21 bp shorter than normal) in *Dendrobium* (Fig 3). Within *Cymbidium*, which is the only genus of orchids with complete plastomes of several species available, different *ndh* genes have been pseudogenized in different lineages, while other *ndh* genes retain full reading frames in different lineages (Figs 3 and 5), all of which suggests a more recent loss of the *ndh* complex in the ancestor of *Cymbidium* and/or partial retention of some loci in the genus. The complex patterns revealed here suggest that further complete plastome sequencing of other major lineages among the earlier-diverging lineages of Orchidaceae, including *Neuwiedia* and Vanilloideae may uncover additional losses and/or retention of the *ndh* gene family.

Although it is perhaps to be expected that the *ndh* loci would be lost independently from distantly related achlorophyllous orchids such as *Rhizanthella gardneri*, *Neottia nidus-avis*, and some *Corallorhiza*, the homoplastic loss of the *ndh* family from photosynthetic Orchidaceae (*Phragmipedium*, *Phalaenopsis*, etc.) is surprising. This repeated loss may be explained by the transfer of *ndh* gene expression to the nuclear genome, as has occurred occasionally for some plastome genes in other angiosperms [13]. Chang *et al*. [26] found in-frame sequences of *ndhA*, *ndhF*, and *ndhH* in the nuclear genome of *Phalaenopsis aphrodite* and concluded that the ancestral functional *ndh* copies of the plastome had been transferred to the nuclear genome. The first nuclear genome sequence of an orchid, *Phalaenopsis equestris*, has recently been published [66], but no full-length, in-frame *ndh* sequences are apparent among the coding sequences in the published sequence, which is consistent with the complete loss of chlororespiratory function in at least some photosynthetic orchids. However, several small to medium-sized ORFs have regions of 60–500 bp in length with high nucleotide sequence similarity to parts of some *ndh* loci (specifically, *P*. *equestris* CDS loci PEQU_01618, PEQU_05946, and PEQU_06248 for *ndhB*, PEQU_06901 for *ndhC*, and PEQU_02231, PEQU_21647, and PEQU_37741 for *ndhD*, PEQU_31033, PEQU_35083, and PEQU_41119 for *ndhF*, and PEQU_02231 for *ndhK*), suggesting that perhaps there have been past transfers of some *ndh* loci to other parts of the genome. Moreover, partial transcripts containing pseudogenized portions of most *ndh* loci are also present in the *P*. *equestris* transcriptome (GenBank TSA accession number GDHJ00000000.1), including portions of *ndhB* (GDHJ01026700.1 and GDHJ01034679.1), *ndhC* (GDHJ01007613.1 and GDHJ01031579.1), *ndhD* (GDHJ01051652.1|, GDHJ01051149.1, and GDHJ01018088.1), *ndhF* (GDHJ01055803.1), *ndhG* (GDHJ01011109.1), *ndhI* (GDHJ01011109.1), *ndhJ* (GDHJ01007613.1) and *ndhK* (GDHJ01007613.1; note that this transcript contains portions of *ndhC*, *ndhJ*, and *ndhK*, which form a cistron in angiosperm plastomes). Partial and complete transcripts of some *ndh* loci have also been detected in whole-cell transcriptomes of orchids that have lost these loci in the plastomes, including *Vanilla planifolia*, *Paphiopedilum armeniacum*, and *Erycina pusilla*, and fragments of *ndh* loci have been found in the mitochondrial genomes of *E*. *pusilla* [8]. In any case, the expression patterns of the *ndh* loci have not been assessed in any orchid, regardless of the presence or absence of complete or partial *ndh* ORFs in the plastome or elsewhere, and thus it is not clear whether the *ndh* genes are essential for the growth of photosynthetic orchids. Additional genome and transcriptome sequencing of orchids in key phylogenetic positions may help clarify expression patterns and possible transfers to the nucleus and/or mitochondrion.

A putative transfer of all *ndh* loci to the nucleus in the common ancestor of Vanilloideae, Cypripedioideae, Epidendroideae, and Orchidoideae could help explain the serial loss of the *ndh* genes across multiple orchid lineages, because the plastid copies would become unnecessary and might be expected to degrade through time. It does not explain, however, why the *ndh* loci were retained in *Cypripedium*, *Goodyera*, *Habenaria*, *Masdevallia*, *Sobralia*, *Elleanthus*, and *Calanthe*. Across angiosperms, the loss of the *ndh* loci appears strongly correlated with changes in trophic status away from full autotrophy, resulting in the loss of chlororespiratory function (e.g., [38, 67, 68]). *Cypripedium*, *Goodyera*, *Habenaria*, and *Calanthe* are terrestrial whereas *Masdevallia*, *Sobralia*, and *Elleanthus* are epiphytes or facultative terrestrials, but collectively these seven genera are unremarkable both ecologically and physiologically compared to their *ndh-*lacking photosynthetic relatives. All orchids are parasitic on fungi for germination and establishment [69], but degree of parasitism and nutritional modes of adult plants vary widely among and within taxa. Terrestrial orchid species display a continuum of nutritional modes from autotrophic to mixotrophic to achlorophyllous mycoheterotrophic; orchid plants usually parasitize ectomycorrhizal associates of nearby forest trees or saprotrophs [70, 71]. Mature, flowering albino (nongreen) individuals can be found in various normally green species, including *Cephalanthera*, *Limodorum*, and *Epipactis* [72–74]. In *Cymbidium*, mycoheterotrophic plants evolved after the establishment of mixotrophic nutrition rather than directly from autotrophic ancestors, suggesting that this course would be one of the principal patterns in the evolution of mycoheterotrophic species [75]. Epiphytic orchids such as *Masdevallia* and *Sobralia* have never been documented to include nongreen mycoheterotrophic species or individuals (W. M. Whitten, personal observation) and probably are fully autotrophic at maturity, since they can be cultivated on inorganic substrates and treated with fungicides without detriment. It may be possible that *ndh* gene deletion in orchid plastomes was initially associated with the evolutionary appearance of the unusual mycoparasitism in orchids in some way, although no causal relationship can be confirmed with the data at hand.

The loss of the *ndh* loci has likely also led to additional structural changes to orchid plastomes, as evidenced by the apparent strong correlation between *ndhF* deletion and the instability of the IR/SSC junction in Orchidaceae. In all orchid plastomes that have full (*Cypripedium*, *Goodyera*, *Habenaria*, *Sobralia*, *Elleanthus*, *Canlanthe* and *Masdevallia*) or nearly complete (*Cymbidium*) *ndhF* ORFs, approximately 1 kb of the 5′ end of *ycf1* is located within the IR, and the 3′ end of *ndhF* gene lies very near the SSC/IR junction, as is typical of most angiosperm plastomes (Fig 5, although this is not seen in one of the two outgroup plastomes, *Eustrephus*). In contrast, in most orchid plastomes where *ndhF* is completely deleted (e.g., *Phragmipedium*, *Neottia*, *Corallorhiza*, *Cattleya*, *Phalaenopsis*, *Erycina* and *Oncidium*) or is severely truncated (*Dendrobium*), the 5′ end of the *ycf1* gene occupies varying positions in or out of the IR, with the portion of the gene within the IR usually significantly shortened compared to plastomes that contain *ndhF* (Fig 5). The *ndhF-*lacking *Paphiopedilum* and *Vanilla* have experienced even more complicated shifts of the IR/SSC boundaries.

Among other lineages of *ndh-*lacking angiosperms, interpreting IR boundary shifts is severely complicated by extensive gene loss due to loss of photosynthesis, loss of the IR, and/or extensive structural rearrangements [40–46]. The *ndh-*lacking plastome with by far the best comparability to those of orchids is that of *Najas flexilis* (Hydrocharitaceae) [47]. Like orchids, *Najas* is photosynthetic and has a plastome that possesses the ancestral angiosperm gene order and has all genes present and functional except for the *ndh* loci; importantly, *ndhF* is completely absent in *Najas*. Very similar to what is observed in orchids with *ndhF* loss, the plastome of *Najas* has an IR/LSC boundary near the start (36 bp upstream) of *rps19* but has experienced a significant shift of the IR/SSC boundary such that *ycf1* is entirely included within the IR [47]. Given these shared patterns of conserved IR/LSC and shifted IR/SSC boundaries in *Najas* and orchids, it seems likely that the loss (and, importantly, not simply the pseudogenization) of *ndh* loci, and *ndhF* in particular, is responsible for the destabilization of the IR/SSC junctions in these plastomes.

## Conclusions

The complex pattern of *ndh* gene loss and retention across Orchidaceae, combined with the unusual trophic strategies of orchids and shifts in the typical plastome IR boundaries, make this family an attractive system for understanding both *ndh* gene loss/transfer and IR boundary movement in angiosperms. The *ndh* gene family has experienced extensive loss in other angiosperm groups as well, typically associated with changes in trophic status and/or extensive plastome structural rearrangements. However, no other angiosperm lineage is known to have multiple, asynchronous *ndh* gene family losses coupled with apparent retention in independent photosynthetic lineages. Furthermore, the apparent correlation between *ndhF* presence/absence and the position of the IR/SSC boundary suggests a potential role for this gene in stabilizing the IR/SSC boundary, which is worth exploring further in additional orchid and non-orchid plastomes.

## Supporting Information

S1 Table GenBank accession information. (DOCX)

S2 Table List of genes found in the plastomes of the seven orchids sequenced for this study. (DOCX)
