# Supplementary material for: Seven New Complete Plastome Sequences Reveal Rampant Independent Loss of the ndh Gene Family across Orchids and Associated Instability of the Inverted Repeat/Small Single-Copy Region Boundaries
Source: PLoS One. 2015 Nov 11;10(11):e0142215. doi: 10.1371/journal.pone.0142215 (PMC4641739; doi:10.1371/journal.pone.0142215)
Supplement: S1 Table — (DOCX) [file pone.0142215.s001.docx]

**S1 Table.** GenBank accession information for the 124 plastomes used for the molecular phylogenetic analyses in this study. Newly sequenced plastomes are highlighted in blue.

| Familes | Taxa | GenBank | Familes | Taxa | GenBank |
| --- | --- | --- | --- | --- | --- |
| outgroups | *Amborella trichopoda* | NC005086 | Orchidaceae | *Vanilla planifolia* | KJ566306 |
|  | *Chloranthus spicatus* | NC009598 | Dasypogonaceae | *Dasypogon bromeliifolius* | NC020367 |
|  | *Illicium oligandrum* | NC009600 | Dioscoreaceae | *Dioscorea elephantipes* | NC009601 |
|  | *Nuphar advena* | NC008788 | Alstroemeriaceae | *Alstroemeria aurea* | KC968876 |
|  | *Nymphaea alba* | NC006050 | Alstroemeriaceae | *Bomarea edulis* | NC025306 |
|  | *Trithuria inconspicua* | NC020372 | Liliaceae | *Fritillaria taipaiensis* | NC023247 |
| Acoraceae | *Acorus americanus* | NC010093 | Liliaceae | *Lilium longiflorum* | KC968977 |
| Acoraceae | *Acorus calamus* | NC007407 | Liliales | *Chionographis japonica* | KF951065 |
| Araceae | *Colocasia esculenta* | NC016753 | Liliales | *Veratrum patulum* | NC022715 |
| Araceae | *Lemna minor* | NC010109 | Smilacaceae | *Smilax china* | HM536959 |
| Araceae | *Spirodela polyrhiza* | NC015891 | Petrosaviaceae | *Petrosavia stellaris* | NC023356 |
| Araceae | *Wolffia australiana* | NC015899 | Poaceae | *Anomochloa marantoidea* | NC014062 |
| Araceae | *Wolffiella lingulata* | NC015894 | Poaceae | *Acidosasa purpurea* | NC015820 |
| Hydrocharitaceae | *Elodea canadensis* | NC018541 | Poaceae | *Arundinaria appalachiana* | NC023934 |
| Hydrocharitaceae | *Najas flexilis* | NC021936 | Poaceae | *Arundinaria gigantea* | NC020341 |
| Arecaceae | *Cocos nucifera* | NC022417 | Poaceae | *Arundinaria tecta* | NC023935 |
| Arecaceae | *Elaeis guineensis* | NC017602 | Poaceae | *Ferrocalamus rimosivaginus* | NC015831 |
| Arecaceae | *Calamus caryotoides* | NC020365 | Poaceae | *Indocalamus longiauritus* | NC015803 |
| Arecaceae | *Pseudophoenix vinifera* | NC020364 | Poaceae | *Phyllostachys edulis* | NC015817 |
| Arecaceae | *Bismarckia nobilis* | NC020366 | Poaceae | *Phyllostachys nigra var. henonis* | NC015826 |
| Arecaceae | *Phoenix dactylifera* | NC013991 | Poaceae | *Phyllostachys propinqua* | NC016699 |
| Asparagaceae | *Eustrephus latifolius* | KM233639 | Poaceae | *Bambusa emeiensis* | NC015830 |
| Orchidaceae | *Apostasia wallichii* | HQ180402-HQ183419 | Poaceae | *Bambusa oldhamii* | NC012927 |
| Orchidaceae | *Cypripedium formosanum* | KJ501998 | Poaceae | *Dendrocalamus latiflorus* | NC013088 |
| Orchidaceae | *Cypripedium japonicum* | KJ625630 | Poaceae | *Leersia tisserantii* | NC016677 |
| Orchidaceae | *Cypripedium macranthos* | NC024421 | Poaceae | *Oryza meridionalis* | NC016927 |
| Orchidaceae | *Paphiopedilum armeniacum* 1 | KT388109 | Poaceae | *Oryza rufipogon* | NC017835 |
| Orchidaceae | *Paphiopedilum armeniacum* 2 | KJ566307 | Poaceae | *Oryza sativa Japonica Group* | NC001320 |
| Orchidaceae | *Paphiopedilum niveum* | KJ524105 | Poaceae | *Rhynchoryza subulata* | NC016718 |
| Orchidaceae | *Phragmipedium longifolium* | KM032625 | Poaceae | *Brachypodium distachyon* | NC011032 |
| Orchidaceae | *Corallorhiza bulbosa* | NC025659 | Poaceae | *Agrostis stolonifera* | NC008591 |
| Orchidaceae | *Corallorhiza macrantha* | NC025660 | Poaceae | *Deschampsia antarctica* | NC023533 |
| Orchidaceae | *Corallorhiza mertensiana* | NC025661 | Poaceae | *Festuca altissima* | NC019648 |
| Orchidaceae | *Corallorhiza odontorhiza* | NC025664 | Poaceae | *Festuca arundinacea* | NC011713 |
| Orchidaceae | *Corallorhiza striata* var*. vreelandii* | JX087681 | Poaceae | *Festuca ovina* | NC019649 |
| Orchidaceae | *Corallorhiza trifida* | NC025662 | Poaceae | *Festuca pratensis* | NC019650 |
| Orchidaceae | *Corallorhiza wisteriana* | NC025663 | Poaceae | *Lolium multiflorum* | NC019651 |
| Orchidaceae | *Calanthe triplicata* | NC024544 | Poaceae | *Lolium perenne* | NC009950 |
| Orchidaceae | *Cymbidium aloifolium* | NC021429 | Poaceae | *Aegilops cylindrica* | NC023096 |
| Orchidaceae | *Cymbidium mannii* | NC021433 | Poaceae | *Aegilops geniculata* | NC023097 |
| Orchidaceae | *Cymbidium sinense* | NC021430 | Poaceae | *Aegilops speltoides* | NC022135 |
| Orchidaceae | *Cymbidium tortisepalum* | NC021431 | Poaceae | *Aegilops tauschii* | NC022133 |
| Orchidaceae | *Cymbidium tracyanum* | NC021432 | Poaceae | *Hordeum vulgare subsp. vulgare* | NC008590 |
| Ochidaceae | *Dendrobium officinale* | NC024019 | Poaceae | *Secale cereale* | NC021761 |
| Orchidaceae | *Masdevallia coccinea* | KP205432 | Poaceae | *Triticum aestivum* | NC002762 |
| Orchidaceae | *Masdevallia picturata* | KJ566305 | Poaceae | *Triticum monococcum* | NC021760 |
| Orchidaceae | *Cattleya crispata* | NC026568 | Poaceae | *Triticum urartu* | NC021762 |
| Orchidaceae | *Epipogium aphyllum* | NC026449 | Poaceae | *Phragmites australis* | NC021372 |
| Orchidaceae | *Epipogium roseum* | KJ946455 | Poaceae | *Coix lacryma-jobi* | NC013273 |
| Orchidaceae | *Erycina pusilla* | NC018114 | Poaceae | *Saccharum hybrid cultivar NCo 310* | NC006084 |
| Orchidaceae | *Oncidium* Gower Ramsey | NC014056 | Poaceae | *Saccharum hybrid cultivar SP80-3280* | NC005878 |
| Orchidaceae | *Oncidium sphacelatum* | KM032624 | Poaceae | *Sorghum bicolor* | NC008602 |
| Orchidaceae | *Neottia nidusavis* | NC016471 | Poaceae | *Sorghum timorense* | NC023800 |
| Orchidaceae | *Elleanthus sodiroi* | KR260986 | Poaceae | *Zea mays* | NC001666 |
| Orchidaceae | *Sobralia callosa* | KM032623 | Poaceae | *Panicum virgatum* | NC015990 |
| Orchidaceae | *Sobralia* aff. *bouchei* | KT388108 | Poaceae | *Setaria italica* | NC022850 |
| Orchidaceae | *Phalaenopsis aphrodite* subsp*. formosana* | NC007499 | Poaceae | *Pharus lappulaceus* | NC015990 |
| Orchidaceae | *Phalaenopsis equestris* | NC017609 | Poaceae | *Pharus latifolius* | NC023245 |
| Orchidaceae | *Phalaenopsis* hybrid cultivar | KJ944326 | Poaceae | *Puelia olyriformis* | NC023449 |
| Orchidaceae | *Goodyera fumata* | KJ501999 | Typhaceae | *Typha latifolia* | NC013823 |
| Orchidaceae | *Rhizanthella gardneri* | NC014874 | Heliconiaceae | *Heliconia collinsiana* | NC020362 |
| Orchidaceae | *Habenaria pantlingiana* | KJ524104 | Zingiberaceae | *Zingiber spectabile* | NC020363 |
