# Supplementary material for: Seven New Complete Plastome Sequences Reveal Rampant Independent Loss of the ndh Gene Family across Orchids and Associated Instability of the Inverted Repeat/Small Single-Copy Region Boundaries
Source: PLoS One. 2015 Nov 11;10(11):e0142215. doi: 10.1371/journal.pone.0142215 (PMC4641739; doi:10.1371/journal.pone.0142215)
Supplement: S2 Table — (DOCX) [file pone.0142215.s002.docx]

**S2 Table**. List of genes found in the plastomes of the seven orchids sequenced for this study.

| **Functional group** | | **Present in all seven orchids** | ***S. callosa,***  ***S.* aff*. bouchei,***  ***E. sodiroi,***  ***M. coccinea*** | ***O. sphacelatum*** | ***P. armeniacum*** | ***P. longifolium*** |
| --- | --- | --- | --- | --- | --- | --- |
| **RNA genes** | Ribosomal RNAs | *rrn4.5*(x2)*, rrn5*(x2)*, rrn16*(x2)*, rrn23*(x2) |  |  |  |  |
|  | Transfer RNAs | *trnA-*UGC^a^(x2)*, trnC-*GCA*, trnD-*GUC*, trnE-*UUC*, trnF-*GAA*, trnfM-*CAU*, trnG-*GCC*, trnG-*UCC^a^*, trnH-*GUG(x2)*, trnI-*CAU(x2)*, trnI-*GAU^a^(x2)*, trnK-*UUU^a^*, trnL-*CAA(x2)*, trnL-*UAA^a^*, trnL-*UAG*, trnM-*CAU*, trnN-*GUU(x2)*, trnP-*UGG*, trnQ-*UUG*, trnR-*ACG(x2)*, trnR-*UCU*, trnS-*GCU*, trnS-*GGA*, trnS-*UGA*, trnT-*GGU*, trnT-*UGU*, trnV-*GAC(x2)*, trnV-*UAC^a^*, trnW-*CCA*, trnY-*GUA |  |  |  |  |
| **Protein genes** | Photosynthesis |  |  |  |  |  |
|  | Photosystem I | *psaA, psaB, psaC, psaI, psaJ* |  |  |  |  |
|  | Photosystem II | *psbA, psbB, psbC, psbD, psbE, psbF, psbH, psbI, psbJ, psbK, psbL, psbM, psbN, psbT, psbZ* |  |  |  |  |
|  | Cytochrome | *petA, petB^a^, petD^a^, petG, petL, petN* |  |  |  |  |
|  | ATP synthase | *atpA, atpB, atpE, atpF^a^, atpH, atpI* |  |  |  |  |
|  | Rubisco | *rbcL* |  |  |  |  |
|  | NADH dehydrogenase |  | *ndhA^a^, ndhB^a^(x2), ndhC, ndhD, ndhE, ndhF, ndhG, ndhH, ndhI, ndhJ, ndhK* | *φndhA φndhB(x2), φndhC, φndhD, ndhE, φndhG, φndhH, φndhI, φndhJ, φndhK* | *φndhB^a^(x2), φndhC, φndhD, φndhJ, φndhK* | *φndhA, φndhB(x2) φndhD, φndhI φndhJ, φndhK* |
|  | ATP-dependent protease subunit P | *clpP^a^* |  |  |  |  |
|  | Chloroplast envelope membrane protein | *cemA* |  |  |  |  |
| **Ribosomal proteins** | large units | *rpl2^a^*(x2)*, rpl14, rpl16^a^, rpl20, rpl22, rpl23*(x2)*, rpl32, rpl33, rpl36* |  |  |  |  |
|  | small units | *rps2, rps3, rps4, rps7*(x2)*, rps8, rps11, rps12^a^*(x2)*, rps14, rps15, rps16, rps18, rps19* |  |  |  |  |
| **Transcription** | RNA polymerase | *rpoA, rpoB, rpoC1^a^, rpoC2* |  |  |  |  |
| **/translation** | Initiation factor | *infA* |  |  |  |  |
|  | Miscellaneous proteins | *accD, ccsA, matK* |  |  |  |  |
|  | Hypothetical proteins & Conserved reading frame | *ycf1, ycf2*(x2)*, ycf3^a^, ycf4* |  |  |  |  |

*(x2): duplicated genes, ^a^: genes having introns, *φ*: pseudogene
